# Materia Medica and Therapeutics; Medical Chemistry; Toxicology and Legal Medicine

**Published:** 1860-11

**Authors:** Emil Fischer


### MATERIA MEDICA AND THERAPEUTICS; MEDICAL CHEMISTRY; TOXICOLOGY AND LEGAL MEDICINE.

BY EMIL FISCHER, M.D.

### MATERIA MEDICA AND THERAPEUTICS.

I.—*On the Employment of Stearate of Iron in the Treatment of Soft or Phagedenic Chancres.* By M. RICORD. (Pharmaceutical Preparations of M. Braille.)

From a communication of M. Calvo, nephew of M. Ricord, we learn that the latter has employed for several months, in the *Hôpital du Midi*, an ointment or plaster of stearate of iron, prepared by M. Braille, pharmaceutist of the institution, to whom we are indebted for this excellent means of dressing soft or phagedenic chancres.

This new preparation, which can be prepared at a low price, and is of easy application, is destined, without doubt, to render great service in all those cases of so serious a character where the phagedenic action seems to resist the numerous means which science directs against it, and continues its destructive march without interruption. Up to the present time, at least, it has fulfilled, in the hands of M. Ricord, all that it seemed to promise, and has become of daily use in his hospital as well as in his private practice.

The *modus faciendi* of these new preparations, as communicated by M. Braille, is the following:—

*Ointment of Stearate of Iron.*—℞. Sulphate of Iron, 500 grammes; Marseilles Soap, 1000 grammes.

Dissolve the sulphate of iron in about 1500 grammes of water, and dissolve the Marseilles soap in an equal quantity of water. On pouring one solution into the other, a whitish-green precipitate is obtained, which is dried, and then melted at a moderate temperature (80° to 84° R.;) add to the melted mass, on cooling, 40 per cent. of essence of lavender, and stir it constantly until it becomes perfectly cold.

*Sparadrap of Stearate of Iron, (Braille's Plaster.)*—℞. Stearate of Iron, q. s., obtained by the process directed above.

Melt it at a moderate temperature, and spread it on muslin like the ordinary sparadrap. This mass gives an adhesive sparadrap, which does not crack like the soaps of lead obtained by double decomposition.—(*Journal de Pharmacie et de Chimie*, June, 1860.)

II.—*Hydrochloric Acid in Chronic Dyspepsia.* By DR. SCHOTTIN, of Dresden.

Dr. Schottin has used hydrochloric acid with great success in cases of chronic dyspepsia. The curative effect of the remedy is attributable to two circumstances: First, it suspends, like other powerful acids, the process of fermentation; and, secondly, it serves to dissolve the proteinaceous compounds, being, to a certain extent, a substitute for the disturbed secretion of the gastric juice; it is, therefore, the most natural remedy. In children who suffer from gastric and intestinal catarrh, the author prescribes the acidum muriaticum dilutum of the Prussian pharmacopœia, in doses of six to fifteen drops, in a mucilaginous mixture, and adds, until the bowels are regulated, a few drops of tincture of opium. He orders the medicine to be taken half an hour after each meal, and confines the patient to a diet of milk and broth. In old age, when the strength of the system is gradually failing, disturbances of digestion are very frequent, the cause of which is to be found, in many instances, solely in a diminished secretion of the gastric juice. A double indication is to be fulfilled in these cases: to arrest the process of fermentation, and to stimulate the stomach, in order to increase the secretion of the gastric juice. Dr. Schottin recommends for this purpose small doses of chloride of sodium and sulphate of quinia, to be followed, a short time afterwards, by sulphuric acid. The chloride of sodium is decomposed by this means into sulphate of soda, and hydrochloric acid. He prescribes ten grains of chloride of sodium and one-third of a grain of sulphate of quinia, to be taken four times a day wrapped up in a wafer, lets the patient drink some water after it, and administers, about five minutes later, eight to twelve drops of the elixir acidum Halleri in half a wineglassful of water.

The dyspepsia of drunkards requires a double dose of chloride of sodium and sulphuric acid. Dr. Schottin attributes the effect of hydrochloric acid in typhus, anæmia, and chlorosis likewise to its property of suspending the process of fermentation within the stomach, and of exciting the deficient secretion of the gastric juice.—(*Archiv der Heilkunde*, I. 2, and *Med. Central Zeitung*, April, 1860.)

### III.—*Hydrocotyle Asiatica in Chronic Eczema.* By M. Hillairet.

The physiological action of this umbelliferous plant manifests itself, according to the author, by symptoms of more or less intense irritation of the gastro-intestinal canal, vomiting, borborygmus, diarrhœa, and loss of appetite, which are, however, of a transient character, and are never accompanied by narcotic symptoms, as stated by Devergie and Lecoq. Contrary to the experience of Boileau, Lépine, and others, the author has found the hydrocotyle Asiatica perfectly useless in lepra tuberculosa, but has administered it successfully in chronic eczema. He reports five cases of this disease, which occurred in his own practice; of these cases two were completely cured, two ameliorated, and one received no benefit from the treatment. He made use of the alcoholico-aqueous extract, which he prescribed in gradually increased doses, amounting to 0·75 and 1·50 grammes during twenty-four hours. In one of the cases, which he reports in full, the cure was established within two months.—(*Gazette des Hôpitaux*, No. 26, 1860.)

### IV.—*Piperin in Intermittent Fever.* By Dr. Meli.

Dr. Meli, of Venice, as the results of numerous experiments, comes to the following conclusions:—1. The febrifuge power of piperin is both energetic and rapid. 2. Its activity is much greater than that of cinchona. 3. It is more convenient than cinchona, and its succedanea, exhibiting a great activity in a very small compass. 4. It neither changes, retards, nor suppresses any secretion or excretion. The alvine dejections are regularized, and the urinary secretion is rendered active.—(*Presse Belge*, No. 22, and *Med. Times and Gazette*, July, 1860.)

V.—*Arsenic in Apoplectic Congestion.* By M. LAMARE-PICQUOT.

M. Lamare-Picquot, Physician to the Honfleur Hospital, as the result of ten years' observation and trial upon between forty and fifty cases, including his own among them, strongly recommends the prolonged use of arsenic as an effectual means of subduing congestion likely to give rise to apoplexy. In very urgent cases, in which hemorrhage seems imminent, he precedes its employment by a moderate venesection, but this is quite exceptional. In proportion to the severity and menacing danger of the case the dose requires to be larger; and although, even after a month, benefit may already result, to be of permanent benefit it will have to be continued for several months. The more urgent the case, the more tolerant does the system become of the arsenic.

The author, regarding apoplexy as consisting essentially in an excessive increase of globules of the blood, employs arsenic as a powerful agent for decreasing these, as well as the plasticity of the blood. It becomes, of course, necessary to assure one's self in a given case of the richness of the blood, for to employ arsenic when the blood is impoverished would be to do mischief. The author has generally found the dose of gr. $\frac{1}{15}$ to $\frac{1}{6}$ per diem sufficient.—(*Bull. de Thérap.* tome lvii., and *Med. Times and Gazette*, July, 1860.)

VI.—*Asarum Europæum a Remedy for the Effects of Drinking.* By DR. SMIRNOFF.

Dr. Smirnoff states that he has become convinced, from repeated trials, that the *asarum Europæum* well deserves the reputation it has obtained in Russia of being an excellent remedy for the effects of drinking.

The influence of a continuous abuse of alcoholic drinks is first exerted locally, but afterwards dyspepsia is produced; and the nutrition and functions of the entire economy, especially of the central portions of the nervous system, becoming interfered with, the blood itself being loaded with an injurious foreign material, the *dyscrasia potatorum* is at last completely established.

The asarum fulfills various indications, acting beneficially on the alimentary canal in those cases in which the digestive powers are so much at fault. Its aromatic principle confers upon it a stomachic power, and regulates the condition of the intestinal discharges, producing vomiting and purging when given in large doses. Its most beneficial action, however, is manifested on the defective appetite, and by its counteracting the invincible longing for alcohol. The horrible sensations with which the drinker awakes in the morning and which compel him to seek temporary and delusive relief from renewed libations, are much blunted and mitigated by means of a glass of strong infusion of asarum and some other nervine—*e.g.* valerian. Its immediate effect is often to produce vomiting and sometimes purging; but the painful sensations at the epigastrium undergo relief, and the appetite becomes invigorated. Persons who have been long habituated to alcoholic drinks cannot, however, have these suddenly suppressed with impunity; and in such cases the author gives the asarum in brandy, applying at the same time a blister or an issue to the pit of the stomach. By this means the normal activity of the stomach becomes excited and the longing for alcohol diminished.

The author, however, cannot agree with those who would still allow a small quantity of spirits to habitual drinkers, even when the morbid desire for it has become appeased. The continuous use of a decoction of asarum, even when it does not succeed in extinguishing the desire for alcohol, always supports the powers of the patient; and it is remarkable in some cases, in which the individuals have been long accustomed to periodical intervals of drunkenness, ending in delirium tremens, how much longer those intervals will become, and how much less likely delirium tremens is to recur. The patients themselves are sometimes surprised at the comparative impunity with which they can continue their drinking. The author prescribes three or four glasses a day of an infusion made with ℥iij of asarum root, ℥i of valerian root, and ℥ss of orange-peel, but he does not state the quantity of water employed. In cases of drunkenness, another formula is composed of decoction of asarum, (made by boiling from ℥ss to ℥j of the root,) ℥vj, tincture of valerian, ʒij to ʒiij, Sydenham's laudanum, gtt. xij, syrup of orange-peel, ℥ss. A tablespoonful of this is taken every two hours. He finds from two to five grains of bismuth, taken four times a day, a valuable adjunct. He has also found the following popular Russian remedy of service in cases of drunkenness :—℞. Ammon. carb., ℥ss; aceti vini, lbj; oxymel scill., ℥ss. Two tablespoonfuls every two hours.—(*Med. Zeit. Russlands*, and *Med. Times*, July, 1860.)

VII.—*On the Employment of Saccharate of Colchicum in the Treatment of Gout and Articular Rheumatism.* By DR. JOYEUX.

From a great number of cases which have come under the author's observation, he draws the following conclusions :—1. That the saccharate of colchicum, prepared with the fresh juice of the flower, is one of the most reliable remedies which the physician can employ in order to combat the symptoms which depend upon the gouty or rheumatic diathesis. 2. That the curative effects of colchicum are not owing to its irritating action upon the alimentary canal, but to the sedative power of the alkaloids which it contains; and that, consequently, it is of advantage to administer it in fractional and gradually increasing doses, so as to avoid its purgative effect.

The saccharate of colchicum employed by M. Joyeux is prepared with 100 grammes of the fresh juice and 500 grammes of sugar, and evaporated to dryness *in vacuo*. He uses also an extract of the juice of colchicum, evaporated *in vacuo*, as an external application, directing it to be rubbed on the painful parts. The saccharate is given in the average dose of four grammes per diem, divided into ten parts, one of which is taken every hour.

"Since I have made use of these preparations," says the author, "I have not met with a single case of gout which did not yield to treatment in two or three days. Acute articular rheumatism disappeared in the space of fifteen to twenty days. In subacute rheumatism, without obtaining an equally satisfying result, I have witnessed a great amelioration. I have found it of advantage, in the majority of cases, to let the patient take, as adjuvant, an infusion of lime-tree blossoms, containing nitre, in the proportion of two grammes to one litre of tea.—(*Gazette Médicale de Strasbourg*, 1860, No. 2, and *Gaz. Hebdom.*, May, 1860.)

### VIII.—*On the Use of Chloride of Iron in Cutaneous Diseases.* By M. DEVERGIE.

From the experiments which the author has made with this remedy in the Hôpital Saint-Louis, he arrived at the following conclusions:—1. The internal administration of chloride of iron is followed by excellent success in purpura hæmorrhagica and purpura simplex. 2. Its internal use is also advantageous in combating the cachectic and anæmic condition which often accompanies certain diseases of the skin, (rhypia, ecthyma cachecticum, impetigo scabida, and atonic ulcers of the leg.) The remedy is given internally in the dose of ten to thirty drops daily, in the form of julep. In active hemorrhage it is less efficient. 3. Chloride of iron, applied externally, may serve, according to the different degree of concentration, as a "modifying" agent in wounds, atonic, scrofulous and syphilitic ulcers, as well as in chronic diseases of the skin attended by secretion, and may therefore be used with great advantage in these affections. 4. The application of chloride of iron in the form of ointment is best suited to the declining stage of secreting diseases of the skin; strong ointments of this kind, however, exercise a modifying influence also upon cutaneous diseases of a scaly character.—(*Bull de Thérap*, lviii., April, 1860.)

### IX.—*Ether as a Remedy for Deafness.*

The attention of the public, both medical and non-medical, has been of late attracted by a fact which, though in its origin and early phases going back some years, is of interest through the publicity given to it by the official journal of the University of Paris, as well as through the daily press.

To state the question briefly:—About the month of August, 1855, a certain Mademoiselle Cléret, a private governess, inhabiting a populous part of Paris, applied to the Minister of Public Instruction for assistance, basing her application, among other grounds, upon her knowledge of a method of causing the deaf and dumb to hear. This method, the discovery of which was accidental, and ot which she had made a successful trial upon some pupils suffering from deafness, after having experienced its efficacy in her own person, consists in the use of sulphuric ether dropped direct into the external auditory canal, at the rate of four, five, six, or eight drops per day. After the application of the remedy for fifteen or twenty days, its use may be suspended some days, and then renewed: it may be continued, if not indefinitely, at least for a very lengthened period.

A commission appointed by the minister, and of which the medical element included M. Lélut as president, M. Béhier as secretary, and the late M. Bérard, was deputed to examine into the state of the children submitted to it by Mademoiselle Cléret. The commission pursued its investigation with the utmost diligence, until upon a sudden the lady was seized with a fearful malady. After having waited, without much hope, an improvement in the mental condition of Mademoiselle Cléret, the commission drew up its report, although the question, necessarily suspended, did not appear susceptible of being brought to a definite conclusion, or to a complete and demonstrative result. It considered, however, that it was its duty to make known such facts as it had witnessed: this it did in the following terms:—

Twenty-nine children have been treated by this lady; all with beneficial results. Two of those brought by her before the commission, and who had been treated by her previously, were completely cured. Seven children have been submitted to the commission previous to any trials being made upon them, and their absolute deafness and dumbness demonstrated by Mademoiselle Cléret; and in all cases, but especially in four, a manifest change has been perceptible after eight or nine months of treatment, and the patients have been able to recognize with great ease various sounds. The reporter to the commission has been careful to add that the most minute precautions were taken to avoid sources of error, and to guard against any illusion that might arise from perception derived through another sense than that of hearing.

The commission wishing to test the means used by Mademoiselle Cléret upon other cases than those exclusively under her own care, deputed one of their number to carry out her plan of treatment upon other patients. Twenty persons were accordingly entrusted to him; most of them were deaf-mute children, but there were also some old men whose hearing was impaired, in some cases upon one side only. In all these a very noticeable effect was produced. It was also found that patients whose sense of hearing had become impaired through typhoid fever were very speedily restored by the same treatment.

In conclusion, the commission state that, with the exception of two or three children, whose deafness and dumbness was attested by authentic certificates, and who now hear well, it has determined nothing but the incomplete results of experiments commenced, but not terminated, which manifest improvement, but nothing more definite.—(*Gazette des Hôpitaux*, 8th May, 1860, and *London Pharmaceutical Journal*, August, 1860.)

X.—*History and Pretensions of Cod-Liver Oil.* Synopsis of a Lecture Delivered by Robert R. McIlvaine, M.D., before the Academy of Medicine of Cincinnati, February 6, 1860. (Reported by J. A. Thacker, M.D., Recording Secretary.)

After the minutes of the last meeting were read and approved, the president announced that the subject for discussion for the evening was, the History and Pretensions of Cod-Liver Oil as an Antidote for the several Diseases for which it is applied.

Dr. Robert R. McIlvaine was here introduced, who, after a few preliminary remarks concerning the Academy, and the nature of its organization, proceeded to discuss the subject as announced.

He said they had met to-night to inquire into the claims and pretensions of cod-liver oil as a therapeutic agent. It was claimed, that at a period previous to Christian civilization, fish-liver oil had been in use as a medicinal agent, and in proof of which, its enthusiastic advocates had quoted as authority the apocryphal books of the Bible, to wit, Tobit, chapter vi. But he would say, for the information of those whose pious scruples forbade them to read the apocrypha, or, for other reasons, that the word oil is not found in the record.

He then spoke of the traditional claims set up for it in Holland and England, from time immemorial, as a popular remedy; but the first who brought it before the notice of the profession was the great Nicholis, in Germany, about the year 1775. The next author of distinction that introduced it to the notice of the profession was Percival, of England, about the year 1785, or, as some say, 1790.

Be this as it may, the curative properties ascribed to it then were very dissimilar to those said to be possessed by it now.

The doctor then passed on to the inquiry as to whether the cod-liver possessed any function peculiar to itself. He spoke of the discovery of the great Bernard, of France, in the liver of the mammalia, and its function of sugar-making.

In his research, however, he discovered that this property is not peculiar to that class, but is also possessed by the galliguassi, fishes, and reptiles. Most of the representatives of each of these families, with the exception of the fresh cod-fish, Bernard had tested; to test this latter was impossible, as the distance from which it could be obtained was too remote for him to make the experiment. Hence the doctor stated, that when leaving France, in August, '59, he prepared himself with the necessary tests, tubes, and apparatus for experiment, in case an opportunity should offer when arriving at the Banks of Newfoundland. On the twenty-fifth of the same month, this opportunity was afforded. On that day, while the ship City of Washington, in which he sailed, was laying too, off the Banks, to send out her news, he procured some fresh codfish from the fishermen, and succeeded in putting beyond controversy the fact that the law that is universal in all animals is not excepted in the case of the codfish.

Here the doctor exhibited the identical sugar to which he had alluded, with specimens of his research since his return to this city, including sugar obtained from the livers of the pike fish, the catfish of the Ohio, the white-fish of the lake, with other specimens obtained from the three great families before alluded to—among them was one from the tortoise, a representative of the reptile family.

This, we believe, is the first demonstrative lecture ever delivered upon the subject in Cincinnati.

He then went on to inquire what were the properties and affinities of cod-liver oil as compared with other oils; for, said he, if it possesses the same ultimate principles as other animal oils, then the claims of its advocates are well.

The doctor then went into an examination of animal oils, quoting from the analyses of gentlemen whose names, he said, were a strong tower, the granite columns in the Esculapian temple, to wit: Chevereul, Lecaun, Pellitier, and Bernard; and, with such names to sustain him in his position, he did not fear to be successfully controverted. These, he said, were his great moral barricades; their demonstrations, like a wall of fire, defensive and offensive.

It has been demonstrated, he said, that the fatty principles derived from the bodies of animals are very analogous, in composition and properties, to the vegetable fixed oils—the latter being employed on account of the expense of the former in Britain and elsewhere. Their ultimate elements are carbon, hydrogen, and oxygen; and most of them, like the fixed oils, consist of two or more definite compounds.

The annexed table, which he repeated, made up from the analyses of the before-mentioned gentlemen, shows the composition of several animal fats, and of their products, reference being had to 100 parts:—

| | Carbon. | Hydrogen. | Oxygen. |
|---|---|---|---|
| Human fat . . . . . . . | 79·000 | 11·416 | 9.584 |
| Oleine of human fat . . . . . | 78·566 | 11·447 | 9·987 |
| Hog's lard . . . . . . . | 79·098 | 11·146 | 9 756 |
| Oleine of lard . . . . . . | 79·030 | 11·422 | 9·548 |
| Mutton suet . . . . . . . | 78·996 | 11·700 | 9·304 |
| Oleine of same . . . . . . | 70·354 | 11·090 | 9·556 |
| Spermaceti . . . . . . . | 79·500 | 11·600 | 8·900 |
| Glycerin . . . . . . . | 40·071 | 8·925 | 51·004 |
| Margarine . . . . . . . . | 74· | 74· | 12· |

| | Carbon. | Hydrogen. | Oxygen. |
|---|---|---|---|
| Cod-liver oil, by Dugal Campbell . . | 80·18 | 13·72 | 58·54 |

The doctor then proceeded to give the composition of a number of vegetable oils, which is exhibited in the following. These analyses are by Liebig, Theinard, Gay-Lussac, Wöhler, Dumas, and Ure.

| | Carbon. | Hydrogen. | Oxygen. |
|---|---|---|---|
| Olive oil . . . . . . . . | 77·213 | 13·360 | 9·427 |
| Camphor . . . . . . . | 79·280 | 10·360 | 10·360 |
| Oil peppermint . . . . . . | 77·300 | 12·600 | 10·100 |
| Oil anise . . . . . . . . | 81·400 | 7·980 | 10·620 |
| Oil cloves . . . . . . . . | 70·020 | 7·420 | 22·560 |
| Oil bitter almonds . . . . . | 79·560 | 5·560 | 14·880 |
| Rosemary . . . . . . . . | 45· | 38· | 2· |

He remarked that there is no essential difference between fat and oil, only one has a greater tendency to the fluid state than the other. Fatty substances, both in the vegetable and animal kingdom, seem to be identical.

He drew the attention of the Academy to the fact that the portion of carbon contained in the oil of the bitter almond was almost the same as in cod-liver oil, the first being 79·56 of carbon, and the second 80·18. This oil, he said, was much pleasanter and far less disgusting than the cod-liver oil.

He also examined into the claims of glycerin, which some were proposing as an appendix to cod-liver oil; he showed its proportions and the fallacy of such pretensions.

The doctor then spoke of the uncertainty of the action of cod-liver oil; that even its advocates assumed that months of use were necessary before any amelioration from its effects could be expected; and, that its action in scrofulous phthisis was null. For these opinions he quoted the high authority of Tauffheil, an author of distinction, who, with Donovan, denies its curative properties any way owing to iodine.

Indeed, the pretensions of the iodine advocates, although not inconsiderable, have themselves failed to make out their case successfully. Though they began as early as 1806, they had not agreed among themselves until 1839, that such an element existed; and now, in our day, no author of distinction claims it as a cause of efficiency. He then quoted the authority of Bretonneau in sustaining him, that the same indications had been fulfilled with the whale oil and common oil of other fishes, as with the cod-liver oil.

He then spoke of the evil effects of cod-liver oil, and in this he was sustained by no ordinary name, as the attention of the profession had been called to it by the great Hobeke, of Belgium, who observed that, by the continued use of it, the pelvic bones had become deformed in women, who, before, had borne children without unusual difficulty. Observe, says he, that the diseases for which it is claimed the most efficient are rachitis and strumous diseases; it is true, that others have claimed efficiency for it in rheumatism and gout, though, in these respects, its advocates have not been decided.

Another name of distinction appears on the list, who supposed he had achieved immortality for himself, by associating his name with it, in the cure of phthisis. To Pereira, of Bordeaux, belongs the honor of introducing it to the profession in this connection. He wrote a book on the subject, presented to the Academy of Science of France, containing numerous supposed cases and professed cures; but his cases, when critically examined, became "small by degrees," and his cures "beautifully less," and the hopes he had inspired in others never realized.

Thus has it been with this so-called potent agent, weeks and months being necessary to demonstrate its supposed efficacy. Let us then be honest, and admit we have not found in this oleaginous body, disgusting and nauseating as it is to the taste, and offensive to the smell, the antidote for our woes that the non-progressive in our profession would have us believe.

He enumerated other authors of this system, and designated them the lights of the hour, the blind leaders of the blind, against whom the verdict of posterity will be, that they are the usurpers of places for the time being, satisfied with indorsing doctrines not demonstrated. They had caused much money to be wasted, and had thereby further impoverished the indigent by their mistaken notion of the subject upon which he had treated, being a remedial agent—a sort of panacea—against which common sense enters its protest.—(*The Druggist*, Cincinnati, August 15, 1860.)

### XI.—*A New Alkaloid in Coca.*

Coca is the name under which the leaves of several species of erythroxylon are and have been known in Peru from time immemorial, and which, especially among the Indians, mixed with a little unslaked lime or wood ashes, are used for chewing. (Vide *North American Med.-Chir. Review*, March, 1860, p. 340.) Numerous and somewhat fabulous accounts are given of their physiological action; as for instance, in "Tschudi's Travels in Peru." A moderate use is said to produce excitement of the functions, to enable the chewer to remain some time without food and to bear the greatest bodily exertions, while an immoderate chewing of coca, like that of opium, frequently becomes an habitual vice, producing all the deleterious symptoms and consequences of narcotics, such as a state of half intoxication, half of drowsiness, with visionary dreams, premature decay, complete apathy, and idiocy. These peculiar symptoms rendered the presence of a narcotic principle very probable, and have induced Professor Wöhler and Dr. Niemann, of Göttingen, to undertake the investigation of the substance. The material was furnished by Dr. Scherzer, the naturalist of the exploring expedition in the Austrian frigate Novara. The examination has so far succeeded, by the usual method for the separation of alkaloids, in eliminating a crystallizable base, *cocaine*, crystallizing in small prisms, devoid of color or odor, slightly soluble in water, more readily in alcohol, and very easily in ether. It possesses a strongly marked alkaline reaction and a bitter taste, and acts in so far peculiarly, as it transiently benumbs, or almost paralyzes the part of the tongue which it touches. It bears some resemblance to atropine in its chemical relations, and forms perfect salts with the acids. It is, however, without action on the eye, and its compound with the chloride of gold is remarkable for forming benzoic acid in large proportion upon being heated. Further experiments will throw light on its physiological properties.—(*Druggists' Circular*, August, 1860.)

### XII.—*Action of Different Medicines on the Mental Faculties.* By PROFESSOR OTTO.

All stimulant and exciting medicines increase the quantity of blood that is sent to the brain. If this quantity exceeds a certain amount, then most of the faculties of the mind become over-excited. Nevertheless the degree of this action is observed to vary a good deal in different cerebral organizations; and it is also found that certain stimulants exercise a peculiar and characteristic influence upon special or individual faculties. Thus ammonia and its preparations, as well as musk, castor, wine, and ether, unquestionably enliven the imaginative powers, and thus serve to render the mind more fertile and creative. The empyreumatic oils are apt to induce a tendency to melancholy and mental hallucinations. Phosphorus acts on the instinct of propagation and increases sexual desire; hence it has often been recommended in cases of impotence. Iodine seems to have a somewhat analogous influence, but then it often diminishes at the same time the energy of the intellectual powers. Cantharides, it is well known, are a direct stimulant of the sexual organs, while camphor tends to moderate and lull the irritability of these parts.

Of the metals, arsenic has a tendency to induce lowness and depression of the spirits, while the preparations of gold serve to elevate and excite them. Mercury is exceeding apt to bring on a morbid sensibility, and an inaptitude for all active occupation.

Of narcotics, opium is found to augment the erotic propensities, as well as the general powers of the intellect, but more especially the imagination. Those who take it in excess are, it is well known, liable to priapism. In smaller doses it enlivens the ideas and induces various hallucinations, so that it may be truly said that, during the stupor which it induces, the mind continues to be awake while the body is asleep. In some persons opium excites inordinate loquacity. Dr. Gregory says that this effect is observed more especially after the use of the muriate of morphia. He noticed this effect in numerous patients, and he then tried the experiment on himself with a similar result. He felt, he tells us, while under the operation, an invincible desire to speak, and possessed, moreover, an unusual fluency of language. Hence he recommends its use to those who may be called upon to address any public assembly, and who have not sufficient confidence in their own unassisted powers.

Other narcotics are observed to act very differently on the brain and its faculties from opium. Belladonna usually impairs the intellectual energies; hyoscyamus renders the person violent, impetuous and ill-mannered; conium dulls and deadens the intellect, and digitalis is decidedly anti-aphrodisiac. Hemp will often induce an inextinguishable gayety of spirits; it enters into the composition of the intoxicating drink which the Indians call *bauss*. The use of the *amanita muscaria* is said to have inspired the Scandinavian warriors with a wild and ferocious courage. Tobacco acts in a very similar manner with opium, even in those persons who are accustomed to its use; almost all smokers assert that it stimulates the powers of the imagination.

If the psychological action of medicines were better known, medical men might be able to vary their exhibition, according to the characters and mental peculiarities of their patients. The treatment of different kinds of monomaniacal derangement also might be much improved, and it is not improbable but that even a favorable change might be wrought on certain vicious and perverse dispositions, which unfortunately resist all attempts at reformation, whether in the way of admonition, reproof, or even of correction.—(*Zeitschrift für die Gesammte Medicin*, and *Medico-Chirurgical Review*, July, 1860.)

---

### Medical Chemistry.

I.—*On the Amount of Water contained in the White and in the Gray Substance of the Brain, and on the Relation of the same to Œdema of the Brain.* By M. Marcé.

M. Etoc Demacy has made the assertion in his thesis (1833) that the anatomical cause of the stupidity of insane persons is œdema of the brain. With reference to this statement the author examined carefully the two substances of the brain, and found at 100° C. the following amount of water in 100 parts:—

| | Gray Substance. | White Substance. |
|---|---|---|
| Man (on an average) | 80 | 70 |
| Sheep | 83·6 | 70 |
| Calf | 85·6 | 69·8 |
| Ox | 92·6 | 63·7 |
| Rabbit | 79·2 | 64 3 |
| Pheasant | 82·7 | 76 9 |
| Owl | 76·2 | 66·7 |

In man, as well as in the other animals, the gray substance contains accordingly more water than the white substance.

The author also endeavored to find out whether the brain could absorb more water than it originally contained. He injected pure water into a healthy brain, and then determined the amount of water it contained; further, he submerged pieces of brain, after weighing them, in water for twenty-four to forty-eight hours and longer, and then weighed them again. He found that the pieces had absorbed fifty per cent. of their weight of water, and that thus thirty parts of solid substance did not correspond any more to seventy, but to one hundred and fifty parts of water. The fact was confirmed by the examination of brains the meninges of which were infiltrated with serum; they were proved to contain more water than the normal brain, particularly in the gray substance, (for instance, eighty-five and ninety per cent.)—(*Journal de la Physiologie*, III. 9, p. 213, 1860.)

### II.—*On Uroglaucine.* By Dr. Kletzinsky, of Vienna.

Dr. Kletzinsky, of Vienna, has demonstrated that *uroglaucine* of Heller, *urocyanine* of Aloys Martin, *cyanourine* or *cyanurine* of Braconnot, is identical with indigo. Dr. Kletzinsky was seven years collecting samples of uroglaucine, and in that time succeeded in getting two drachms of the blue coloring matter of urine, which he submitted to a rigid analysis. This analysis furnished the formula, $C_{16}H_5AZO_2$, which is that of indigo. The identity of the two substances is thus placed beyond doubt.

It is remarkable that, according to the experiments of Kletzinsky, the indigo of the urine perceptibly increases after the administration of even small doses of creosote, or of essence of bitter almonds. These facts are, however, not sufficient to establish its mode of formation in the organism; we only know that it augments, especially under the influence of certain irritations of the spinal cord or of its nerves, and in various diseases of the kidneys, and in certain diseases of the serous membranes, with exudation.—(*Gaz. Hebdom.*, and *St. Louis Med. and Surg. Journal*, September, 1860.)

---

### Toxicology and Legal Medicine.

### I.—*Means of Distinguishing Blood-Stains on an Instrument covered with Rust.* By MM. Lesueur and Ch. Robin.

In cases where it is the question to distinguish a stain of blood from a stain of rust, it may happen either that the stain is extremely small, or that either substance is superposed. In both cases chemical reactions become impossible. It is in a case of this description that MM. Lesueur and Robin recognized a very small quantity of blood, scarcely forming a coat, upon an instrument covered with rust. They scraped off, with the aid of a scalpel and magnifying glass, a small portion of the stain, and allowed it to fall in a drop of solution of sulphate of soda, rendered slightly alkaline by the addition of a small quantity of caustic soda; they then examined it by the microscope, with a magnifying power of 520 diameters. At first the substance appeared entirely homogeneous, but at the end of half an hour, it was very much swelled, and at the end of another half hour, it seemed to be formed of globules, which could be separated from each other by moving the plates of glass upon each other. These globules were recognized as blood-globules of one of the mammalia, and could, therefore, be owing to human blood.—(*Journ. des Conn Med. et Pharm.*, and *Journal de Médecine de Bordeaux*, August, 1860.)

II.—*Experimental Researches on Death by Drowning.* By J. H. S. BEAU.

What is the cause which prevents the free entrance of surrounding fluid into the respiratory passages of those who are drowned? The author proposed this problem; to solve it, he instituted and practiced three series of experiments: he took the important precaution of employing very small dogs, as, wishing to submerge them immediately below the surface of the water, he could observe their movements easily, and with the aid of an assistant maintain them in this position. He observed that the immersion of the natural orifices of respiration is, with animals who drown in ordinary circumstances, the condition from which results, by sympathetic or reflex action, the spasmodic closure of the sphincters or orifices of the respiration, and the arrest of the respiratory movements. As to the very small quantity of frothy water which is found in the bronchial tree, it enters at a single inspiration, made quickly at the moment when the animal is surprised by the immersion. From which it results that death by drowning has the greatest resemblance to that which supervenes in consequence of a tetanic affection of respiration. The paper was referred to a committee of Flourens, Milne Edwards, and Bernard.—(*Gaz. Hebdomadaire*, and *Cleveland Med. Gazette*, September, 1860.)

III.—*On the Influence of strong external Pressure upon the Formation of Suggillations.* By DR. MÖLLER.

The occurrence of a case, in which it was of importance to decide whether the appearance of suggillations could be prevented by strong external pressure, induced the author to make a number of experiments on frogs, rabbits, and cats, in which he put partly single extremities, partly the head and the upper portion of the body between two small boards, which were quickly pressed together by means of screws or an iron weight, and removed after twenty to thirty minutes, the position of the parts being left undisturbed. These experiments proved without doubt, that the formation of suggillations is prevented by strong external pressure. In every place where the bones had resisted and protected a part, or where the pressure had been warded off for a time by violent movements, extravasations had taken place. On the contrary, suggillations were never formed in those parts which exhibited the effects of strong and continued pressure—viz., where the bones were crushed and the soft parts flattened.—(*Casper's Zeitschrift für Gerichtliche Medicin*, XVII. 1, 1860.)

IV.—*Researches on the Dangers presented by Schweinfurt Green, Arsenical Green, or Arsenite of Copper.* By PROF. A. CHEVALLIER. (Pamphlet, 60 pp. 8vo., Paris, 1859.)

M. Chevallier has collected in this treatise most of the facts which demonstrate the dangers of arsenical greens, and the laws and regulations relative to their use. The following index of the paragraphs in which he divides his subject is sufficient to show the different circumstances in which arsenical colors have given rise to accidents: 1. Diseases of workmen who manufacture Schweinfurt green. 2. Sweetmeats colored with Schweinfurt green. 3. Colored paper serving as envelope for sweetmeats, etc. 4. Introduction of arsenite of copper in the preparation of alimentary substances. 5. On the dangers presented by paper-hangings. 6. Colors placed in the hands of infants. 7. Dresses, flowers, bracelets colored with Schweinfurt green. 8. Wafers colored with Schweinfurt green, necessity of forbidding the fabrication. 9. Danger of burning arsenical paper. 10. Dangers of arsenical colors, employed for painting toys, cages, &c.—(*Gazette Hebdomadaire*, June 29, 1860.)

V.—*On the Detection and Estimation of Phosphorus and Phosphorous Acid.* By Prof. Scherer.

Within two years the author had occasion to gather much experience from a number of cases of poisoning of animals, and of two men; also from several attempts of poisoning by phosphorus. In one case, the phosphorus from thirty to forty matches, equivalent to about half a grain, proved fatal to a woman in forty-eight hours. He establishes the presence of phosphorus by Mitscherlich's method, with the modification of filling the apparatus with carbonic acid, generated from a few pieces of calcareous spar introduced into the acid liquid. No luminous vapors are obtained, but little of the phosphorus is oxidized, and if the tube dips into distilled water, this is phosphorescent when agitated in the dark, and its vapor blackens nitrate of silver.

To estimate the phosphorus, the last bottle containing the water is connected with another phial containing either neutral or slightly ammoniacal nitrate of silver, which absorbs all the phosphorous vapors that have not been retained by the water. Any globules of phosphorus which may have been obtained are fused together and weighed; the water is added and then evaporated; the chloride of silver is filtered off: the phosphoric acid, which is contained in the filtrate, is estimated in the usual manner, and calculated for phosphorus.

Very minute portions of phosphorus may be recognized, after first ascertaining the absence of sulphhydric acid, the vapors of which will turn sugar-of-lead paper black, and paper moistened with nitroprusside of sodium blue; papers moistened with nitrate of silver are suspended over the acid liquid, which is gently heated; in the presence of phosphorus, the silver will be reduced with a black color. The papers may now be macerated in chlorine water or aqua regia; the filtrate will, after evaporation, contain phosphoric acid, to be recognized as ammonio-phosphate of magnesia, or as phosphomolybdate of ammonia.

If phosphorus has been wholly or partly converted into phosphorous acid, the residue from the first distillation is heated in Mitscherlich's apparatus with sulphuric acid and pure zinc, until the hydrogen ceases to be contaminated with phosphuretted hydrogen, which is conducted into the silver solution, and estimated as indicated before.—(*Annale d. Chimie und Pharmacie*, cxii. 214–220, and *Am. Journal of Pharmacy*, September, 1860.)

VI.—*Poisoning by a Dress containing Arsenic.* By PROFESSOR BLASIUS.

A young lady, who went to a ball in a dress of light green tarlatan, was attacked, after having danced five sets, by a feeling of heaviness and lameness in the lower extremities, tightness of the chest, vertigo and headache, so as to be obliged to leave the ball. The symptoms gradually ameliorated, but the feeling of lameness in the lower extremities remained until the third day. A particular cause, as tight-lacing, etc., could not be discovered, and suspicion was directed to the green dress, which, on chemical examination, was proved to contain a large amount of arsenic and copper.

Professor Blasius believes that, from a dress which, according to the present fashion, is so extremely wide, enough dust containing arsenic may be raised around the dancing person that its inhalation may give rise to symptoms of arsenical poisoning.—(*Deutsche Klinik*, 1860, 5.)

